# On *Levymanus*, a remarkable new spider genus from Israel, with notes on the Chediminae (Araneae, Palpimanidae)

**DOI:** 10.3897/zookeys.326.5344

**Published:** 2013-08-23

**Authors:** Sergei Zonstein, Yuri M. Marusik

**Affiliations:** 1 Department of Zoology, The George S. Wise Faculty of Life Sciences, Tel-Aviv University, 69978 Tel-Aviv, Israel; 2 Institute for Biological Problems of the North RAS, Portovaya Str. 18, Magadan, Russia; 3 Zoological Museum, University of Turku, FI-20014, Turku, Finland

**Keywords:** Spiders, new taxa, taxonomy, Palearctic, South-Western Asia, Near East

## Abstract

*Levymanus gershomi* **gen. n.** et **sp. n.**, is described from southern Israel. The eye arrangement and structure of the male palp indicate that this genus belongs to Chediminae Simon, 1893. *Levymanus* gen. n. differs from other chedimine genera by its unusually long and slender legs, an elongate body, a unique shape of the bipartite thoracic fovea, reduced leg scopulae, smaller spinnerets, and other characters, which are presumably apomorphic. We propose two taxonomic changes: 1) based on widely spaced lateral eyes the Western African genus *Badia* Roewer, 1961 is transferred from Chediminae to Palpimaninae, and 2) *Fernandezina gyirongensis* Hu & Li, 1987 from China, based on palpal morphology, is transferred to the Asian genus *Steriphopus* Simon, 1887 for a new combination *Steriphopus gyirongensis* (Hu & Li, 1987) **comb. n.**

## Introduction

The Palpimanidae is a relatively small family of araneophagous spiders consisting of 131 species in 15 genera (Platnick 2013), distributed in tropical and sub-tropical zones worldwide and absent only in the Nearctic and Australia. The family was divided by Platnick (1975) into three subfamilies: Palpimaninae Thorell, 1870 (Africa and Eurasia), Otiothopinae Platnick, 1975 (almost entirely Neotropical), and Chediminae Simon, 1893 (mainly Paleotropical). The Chediminae includes taxa with closely spaced or touching lateral eyes (Palpimaninae have widely spaced lateral eyes) and with tegular sclerites (lacking in Otiothopinae). Currently, the Chediminae includes 30 species in 9 genera, three of which are monotypic (Platnick 2013). While studying spiders in Israel we found one species that seems to belong to Chediminae, but which had a peculiar carapace shape (especially the fovea) and strongly reduced scopulae. In order to allocate this taxon, the senior author examined representatives of all available genera referred to Chediminae – *Boagrius* Simon, 1893, *Chedima* Simon, 1873, *Diaphorocellus* Simon, 1893, *Hybosida* Simon, 1898, *Sarascelis* Simon, 1887, *Scelidocteus* Simon, 1907, *Scelidomachus* Pocock, 1899 and *Steriphopus* Simon, 1893 (including the holotypes), as well as some specimens belonging to *Colopaea* Simon, 1893 (Stenochilidae). As a result we concluded that the specimens from southern Israel belonged to a new genus and species. The main goals of this paper are: 1) to diagnose and describe the new genus and species; 2) to discuss the relationships of the new genus; 3) to discuss the questionable position of some taxa within the subfamily Chediminae.

## Material and methods

Specimens of the following spider taxa were studied.

*Boagrius* sp. *aff. incisus* Tullgren, 1910 (Zambia), NMHL.

*Diaphorocellus* sp. (two species from South Africa), MNHN, NHML.

*Chedima purpurae* Simon, 1873 (Morocco), MNHN.

*Colopea* sp. (Vietnam), MNHN.

*Hybosida lesserti* Berland, 1920, MNHN.

*Sarascelis* (six species, including types: *Sarascelis chaperi* Simon, 1887, *Sarascelis junquai* Jézéquel, 1964, *Sarascelis lamtoensis* Jézéquel, 1964, *Sarascelis luteipes* Simon, 1887 and *Sarascelis rebiereae* Jézéquel, 1964, and an undescribed species from Nigeria), MNHN, NHML.

*Scelidocteus* (five species, including types: *Sarascelis baccatus* Simon, 1907, *Sarascelis lamottei* Jézéquel, 1964, *Sarascelis pachypus* Simon, 1907, *Sarascelis ochreatus* Simon, 1907, *Sarascelis vuattouxi* Jézéquel, 1964), *Steriphopus lacertosus* Simon, 1898), MNHN, and two undescribed species from Cameroon, NHML.

*Scelidomachus socotranus* Pocock, 1899, NHML.

*Steriphopus crassipalpis* Thorell, 1895 and *Steriphopus macleayi* (O. Pickard-Cambridge, 1873), NHML.

The holotype and paratypes of the new taxon described here, including SEM mounts and dissected specimens were deposited in the spider collection of Tel-Aviv University, IsraelTAU and in the Zoological Museum of the Moscow State UniversityZMMU.

Photographs were taken using a Zeiss Discovery V20 stereomicroscope with a Canon PowerShot G9 camera, and an Olympus SZX16 stereomicroscope with an Olympus E-520 camera, and prepared using the CombineZP software. Scanning electron micrographs were made using the SEM JEOL JSM-5200 scanning microscope at the Zoological Museum, University of Turku, Finland. Photographs of landscapes showing the surroundings of the type locality were taken by Vasiliy Kravchenko. Background maps were obtained from the public internet source http://www.maps-for-free.com. Measurements were made to an accuracy of 0.01 mm. Lengths of leg and palp segments were measured on the dorsal side, from the midpoint of the anterior margin to the midpoint of the posterior margin. All measurements are given in millimetres.

Abbreviations used are as follows. *Eyes*: ALE – anterior lateral, AME – anterior median, PLE – posterior lateral, PME – posterior median; *Spinnerets*: ALS – anterior lateral, PLS – posterior lateral, PMS – posterior median; Is – inframamillar scutum; *Bulb details*: Co – conical outgrowth; Em – embolus; Eo – opening of embolus; Ed – embolic division; La – lamella; Tf – tegular fovea, Tp – tegular process; *Palp and leg structures*: Cs – cymbial scopula, Mc – metatarsal comb, On – onychium, Sh – spatulate hairs, Ts – tarsal scopula. Arrows indicate the elevated posterior rim of the carapace, separate small scuta of the abdomen and claw teeth.

Other used institutional acronyms are: MNHN – Muséum national d’Histoire naturelle, Paris, France; NHML – The Natural History Museum, London, UK; SMF – Senckenberg-Museum (Senckenberg Forschungsinstitut und Naturmuseum), Frankfurt am Main, Germany.

## Taxonomy

### Levymanus gen. n.

http://zoobank.org/BFC42358-F98D-4B14-AE8B-72173B265D9A

http://species-id.net/wiki/Levymanus

#### Type species.

*Levymanus gershomi* sp. n., by monotypy.

#### Etymology.

Both the generic name and the specific epithet are given in honour and memory of Gershom Levy (1937–2009), the prominent Israeli arachnologist, for his immense contribution to Israeli and Near East arachnological research. The gender is masculine.

#### Diagnosis.

In general appearance, especially by the elongate body and the extended dorsal abdominal scutum, *Levymanus* gen. n. resembles the otiothopine genus *Fernandezina* Birabén, 1951 (cf. Platnick 1975, fig. 80; Grismado 2002, fig. 1; Grismado and Ramírez 2008, fig. 4; Piacentini et al. 2013, fig. 5a–f), but can be easily distinguished from it by the presence of the accessory structures in the male palp, accompanying the embolus (Figs 38–48), characteristic for the Chediminae, but absent in the Otiothopinae. Both males and females belonging to the new genus are easily distinguishable from other palpimanids due to the characteristic bipartite thoracic fovea (Figs 10, 12).

#### Description.

Small bicolored chedimine palpimanids with body length 2.0–2.5 in males and 2.5–3.0 in females; legs and abdomen without pattern. Carapace with corrugated cuticle, diamond-oval in dorsal view, narrowed anteriorly and posteriorly. Cephalic part somewhat raised behind eye area – slightly in males, and more noticeably in females. Thoracic fovea short and bipartite, with two separate sulci located side by side; posterior edge of carapace slightly raised (Figs 14–15). Eight eyes. ALE largest, about four to five times larger than other eyes, which are subaequal in size. ALE and PLE almost touching each other. PME widely spaced from each other, as well as from AMEs and from PLEs. Clypeus about two times higher than AME diameter. Chilum inconspicuous. Chelicerae small, equal in length with clypeus; stridulatory ridges absent; cheliceral furrow without true or peg teeth. Sternum shield-like with fine reticulation; labium about as broad at base as it is long. Prosoma posteriorly with short paired triangular extentions and narrow tubular structure (Figs 11, 13) entering pedicel tube of abdomen (= scutopetiolar apparatus *sensu* Saaristo and Marusik (2008)). Palps relatively short; legs I–IV long and slender. Leg formula: 1=4,2,3. Femora with well-developed scale-like microsculpture on the cuticle; scales weakly developed on patella and other segments. Femur I rather long and moderately swollen; patella very long (longer that tibia). Tibia and metatarsus I with weakly developed prolateral scopula. Leg tarsi slightly curved and ascopulate. Claw tufts weakly developed. Leg tarsi with two narrow and dentate claws. Abdominal scuta conforming a rather long and corrugated pedicel tube; dorsal portion of scutum with irregular posterior margin. Scutum in male larger than in female, its dorsal part longer than the ventral in the male (Fig. 15) and ventral and dorsal parts subequal in the female (Fig. 14). Although the dorsal and ventral parts of the scutum are fused, the seams are clearly visible in females (Fig. 12) and the dorsal part is rather narrow. Spinneret group very small (Fig. 34). Sclerotised ring encircling spinnerets present but weakly raised. AMS tiny and domed (Fig. 32); PMS and PLS reduced to a few sessile spigots in females, absent in males. Male palp: patella very small without dorsal process; tibia enlarged (swollen); both articles sub-globular. Cymbium moderately long lacking processes and with clusters of setae: a bunch of setae near prolateral base (Figs 43, 48), and larger setae on retrolateral side (Figs 44, 48). Tegulum with relatively short embolic division (Figs 41–46). Female genitalia with large spermathecae and with short and distal seminal ducts (Figs 49, 50).

#### Species included.

*Levymanus gershomi* sp. n.

#### Distribution.

The genus is currently known only from the Arava Valley, Israel.

### Levymanus gershomi sp. n.

http://zoobank.org/BBABE77C-15AE-494D-8A61-6309D6541855

http://species-id.net/wiki/Levymanus_gershomi

Figs 1–2 , 10 –50

#### Types.

Male holotype, 3 ♂ and 2 ♀ paratypes from the vicinity of Qetura (Ktura), Arava Valley, Israel (29°58'N, 35°03'E), 8 May 2003, coll. E. Topel, deposited in TAU (holotype and most paratypes) and ZMMU (few paratypes).

#### Diagnosis.

As for the genus.

#### Description.

Male (holotype): Total length 2.55; carapace, sternum and labium intensive carmine-red; chelicerae, palps (entirely), coxa and femur I light reddish orange, other segments of leg I and entire legs II–IV pale yellowish red; abdomen milk-white with intensive reddish orange dorsal scutum. Carapace (Fig. 10): 1.10 long, 0.76 wide. Diameters of AME, ALE, PLE, PME: 0.10, 0.02, 0.02, 0.02. Interdistances: AME–AME 0.08, ALE–AME 0.05, ALE–PLE <0.01, PLE–PME 0.11, PME–PME 0.14. Chelicerae as shown in Figs 17–18. Sternum (Fig. 11) 0.85 long, 0.65 wide; labium 0.21 long, 0.18 wide at base. Measurements of palp and leg segments as shown in Table 1. Scopulae and tarsal claws as shown in Figs 23, 26–31 and 22, 24, 25, 36, 37 respectively. Scarcely distributed scopular hairs approximately as long as metatarsus and tarsus width (Figs 26–31) At least metatarsus III with comb of setae (Fig. 35). Tarsi I–IV with claw tufts, better developed on tarsi I–II (Figs 22, 24). Tarsal claws I–II with few teeth (Figs 22, 25), III–IV with more and longer teeth (Figs 36, 37). Spinnerets, pedicel tube, and ventral parts of abdominal scutum as shown in Figs 32–34.

**Figures 1–9.**
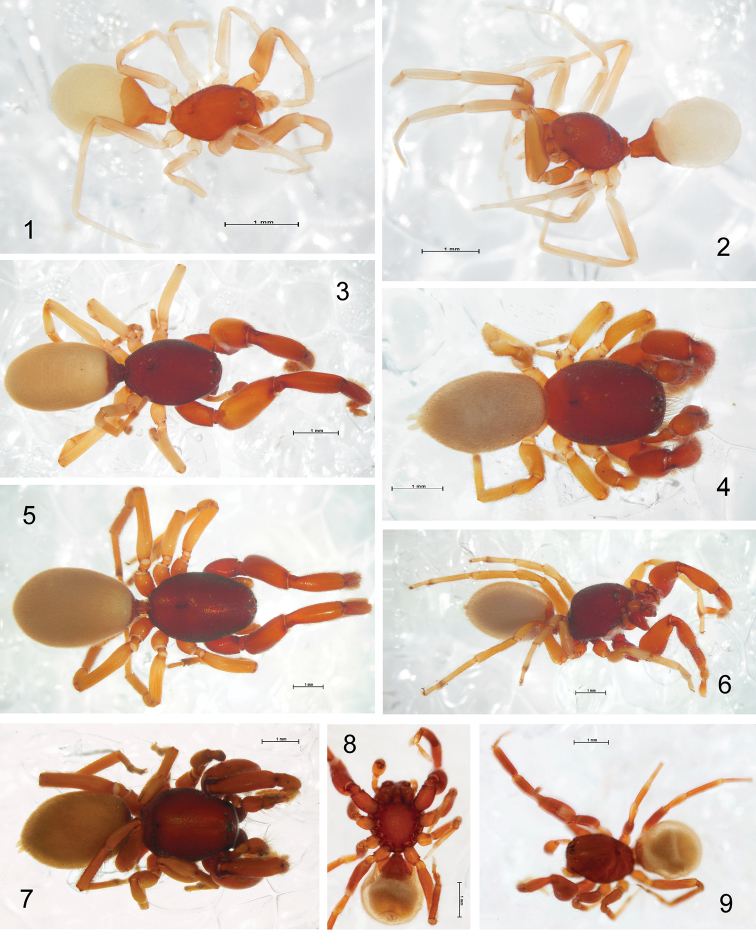
Spiders belonging to the subfamily Chediminae; habitus in lateral (**1, 2, 6**), dorsal (**3–5, 7, 9**) and ventral (**8**) view. **1**, **2** *Levymanus gershomi* sp. n., holotype male and paratype female, respectively **3** *Boagrius* sp. *aff. incisus*, female **4** *Scelidocteus* sp. *aff. vuattouxi*, male **5**, **6** *Sarascelis chaperi*, female and male, respectively **7** *Scelidomachus socotranus*, conspecific male **8**, **9** *Steriphopus macleayi*, holotype male (scale bar = 1 mm).

**Figures 10–13.**
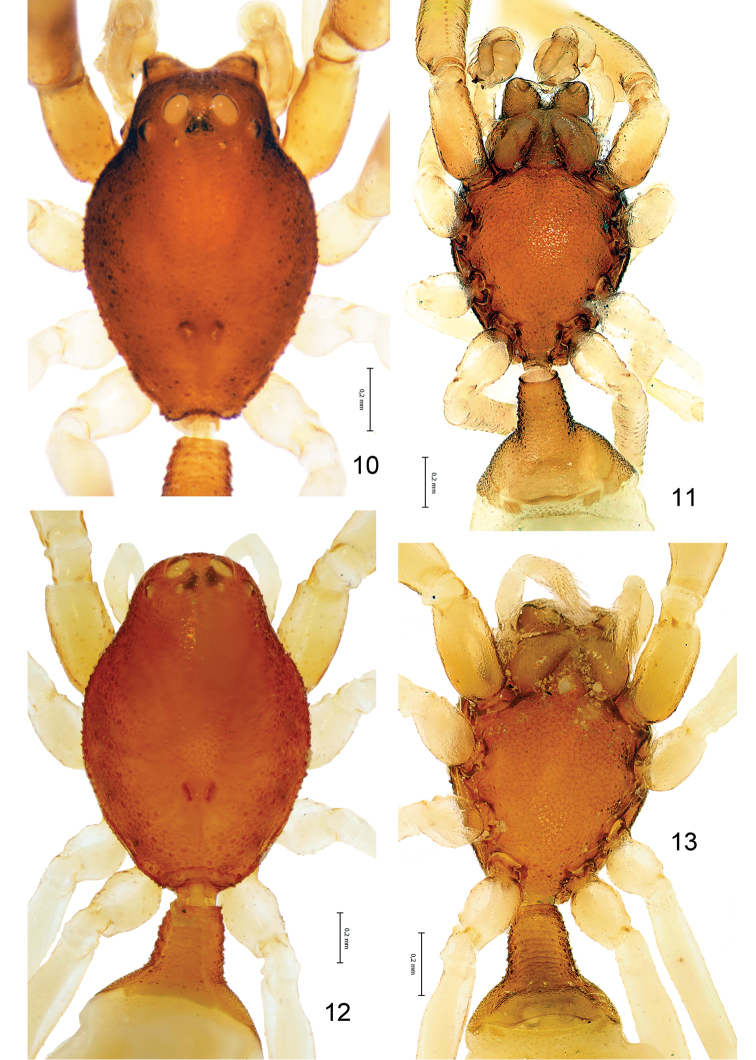
*Levymanus gershomi* sp. n., male (**10**, **11**) and female paratypes (**12**, **13**). **10**, **12** Carapace, dorsal view **11**, **13** Sternum, labium, maxillae and chelicerae; ventral view (scale bar = 0.2 mm).

**Figures 14–16.**
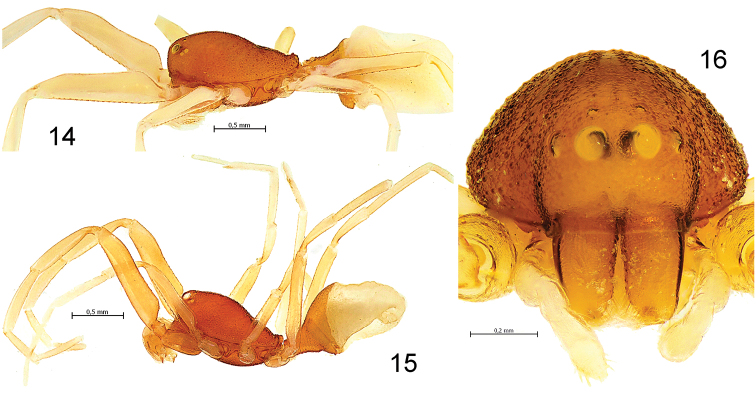
*Levymanus gershomi* sp. n., male (**14**) and female paratype (**15**, **16**). **14**, **15** Habitus, lateral view **16** Carapace and chelicerae, frontal view (scale bar = 0.5 mm for **14**, **15**; 0.2 mm for **16**).

**Figures 17–19.**
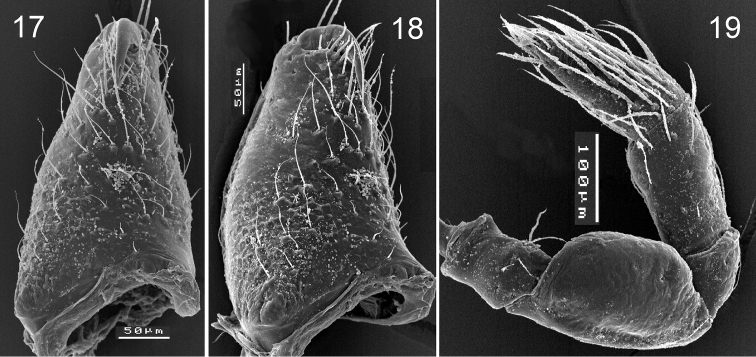
*Levymanus gershomi* sp. n., male (**17**, **18**) and female paratype (**18**). **17**, **18** right chelicera, frontal (**17**) and prolateral (**18**) view **19** right palp, femur to tarsus, retrolateral view. (scale bar = 0.05 mm for **17**, **18**; 0.1 mm for **19**).

**Figures 20–31.**
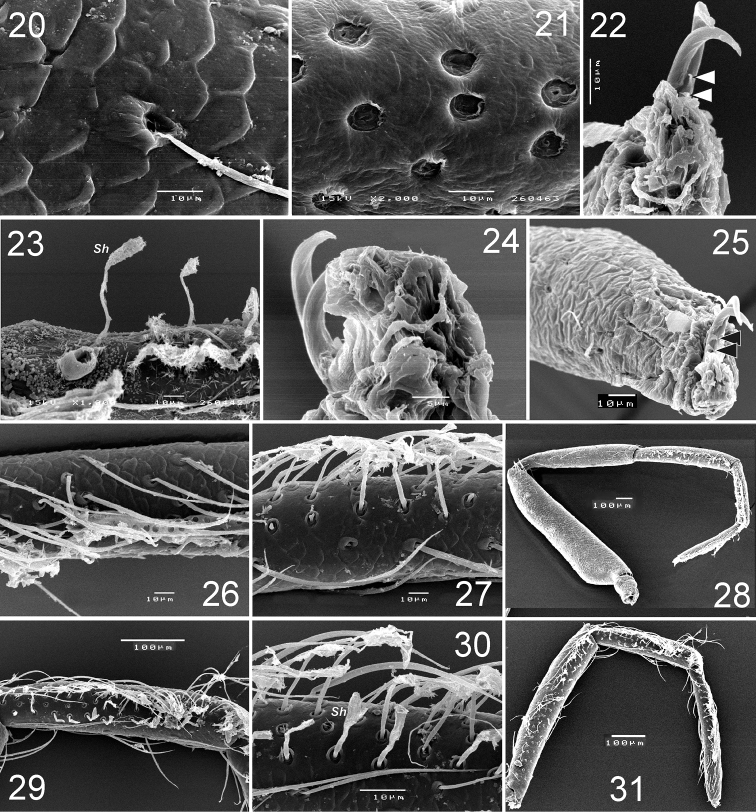
*Levymanus gershomi* sp. n., paratype male, leg I, prolateral view. **20** femur **21** patella **22** tarsus, showing claws with teeth **23** tibia, showing spatulate hair **24** tarsus, showing claws and claw ‘pillow’ **25** tarsus, showing claws and ‘pillow’ **26** tarsus, showing scopula **27** tibia, showing scopula **28** whole leg, showing scopula and enlarged patella **29**, **30** metatarsus I, showing scopula **31** tibia to tarsus, showing scopula (scale bar = 0.1 mm for **20**–**23**, **25**–**27**, **30**; 0.05 mm for **24**; 0.2 mm for **29**, **31**).

**Figures 32–37.**
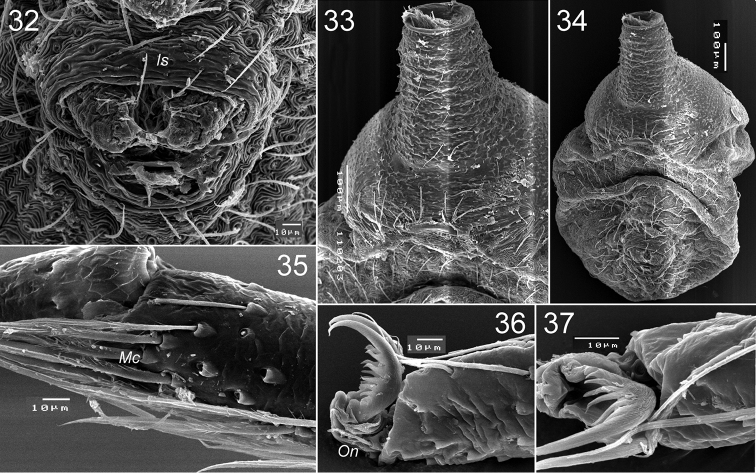
*Levymanus gershomi* sp. n., paratype male. **32** spinnerets, ventral view **33** epigastral scutum, ventral view **34** abdomen, ventral view **35** distal part of metatarsus III, showing metatarsal comb, ventral view **36** distal part of tarsus III, showing onichum and claws, ventral view **37** same, prolateral view (scale bar = 0.01 mm for **32**, **35**–**37**; 0.1 mm for **33**, **34**).

**Figures 38–46.**
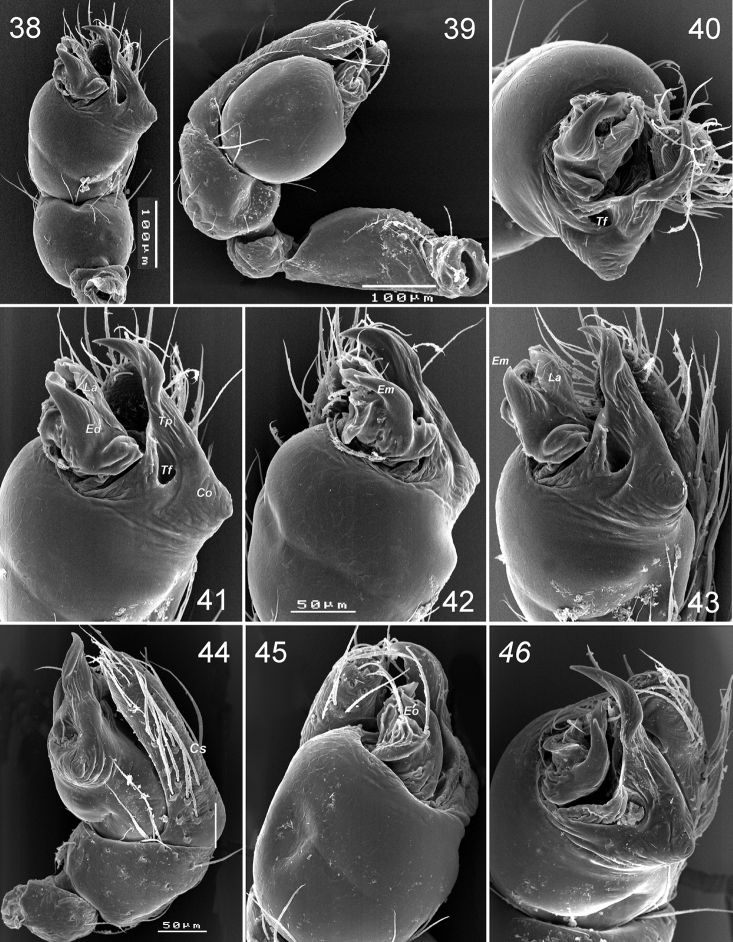
*Levymanus gershomi* sp. n., palp of paratype male **38** palp, ventral view **39** whole palp, prolateral view **40** same, apical view **41** terminal part of bulbus, ventral view **42**, **43** same, prolateral-ventral view **44** same, ventral-retrolateral view **45** palp, retrolateral view **46** same, apical-retrolateral view. **39**, **44**–**46** palp with embolic division sunken into tegulum (scale bar = 0.1 mm for **38**, **39**; 0.05 mm for **40**–**46**).

**Figures 47–50.**
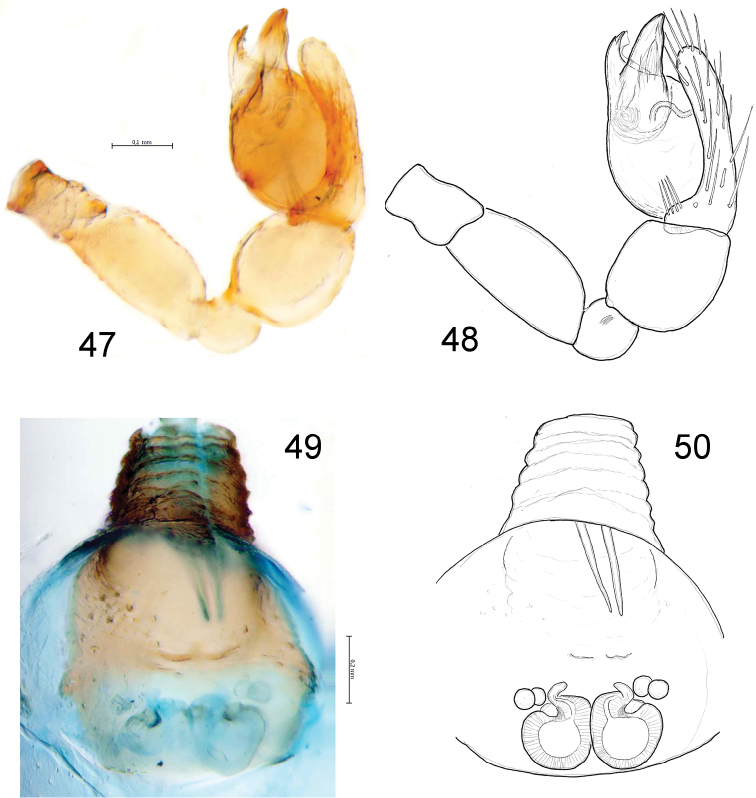
*Levymanus gershomi* sp. n., male (**47**, **48**) and female paratypes (**49**, **50**). **47**, **48** palp, ventral view **49**, **50** epigyne; ventral view (scale bar = 0.2 mm).

Palp (Figs 38–48): femur short and swollen, 2 times longer than wide, subequal in length to tibia and slightly shorter than cymbium. Patella globular. Tibia without apophyses, strongly swollen, 1.5 times longer than wide, 1.3 times wider than femur. Cymbium narrow, shorter than bulb, without outgrowths, with two clusters of hairs on prolateral side (Figs 39, 46). Bulb as wide (in widest part) as long (not counting embolic division and tegular process), tegulum with strong and long retrolateral tegular process (*Tp*); base of process with conical outgrowth (*Co*) and deep furrow (*Tf*); embolus fused with other sclerites of the embolic division (*Ed*). Embolic division, attached to tegulum by a flexible membrane, and bears embolus (*Em*) with retrolateral outgrowth (*Eo*), and lamella (*La*) located between embolus and cymbium.

Female (paratype): coloration as in male. Total length 2.93. Carapace (Fig. 12): 1.34 long, 0.92 wide. Diameters of AME, ALE, PLE, PME: 0.10, 0.03, 0.02, 0.02. Interdistances: AME–AME 0.08, ALE–AME 0.05, ALE–PLE <0.01, PLE–PME 0.10, PME–PME 0.12. Sternum (Fig. 13) 0.94 long, 0.80 wide; labium 0.22 long, 0.24 wide at base. Measurements of palp and leg segments as shown in Table 1. Spermathecae weakly sclerotised, round, touching each other, each spermatheca with a pair of accessory glands (Figs 49, 50). Due to the weak sclerotisation the outline of the spermathecae and their ducts are not very clear.

**Table 1.** *Levymanus gershomi*, gen. et sp. n., palp and leg measurements (in mm). Male holotype and female paratype (in parentheses).

	**Palp**	**Leg I**	**Leg II**	**Leg III**	**Leg IV**
Femur	0.19 (0.29)	1.10 (1.36)	0.78 (0.97)	0.78 (0.82)	1.08 (1.19)
Patella	0.11 (0.12)	0.88 (1.09)	0.53 (0.69)	0.36 (0.49)	0.47 (0.65)
Tibia	0.18 (0.20)	0.71 (0.84)	0.53 (0.73)	0.51 (0.68)	0.69 (0.96)
Metatarsus		0.42 (0.53)	0.47 (0.70)	0.60 (0.64)	0.85 (1.11)
Tarsus	0.27 (0.25)	0.43 (0.55)	0.36 (0.49)	0.32 (0.39)	0.39 (0.46)
Total	0.75 (0.86)	3.54 (4.37)	2.67 (3.58)	2.57 (3.02)	3.48 (4.37)

#### Variability.

Carapace length in males 1.01–1.12, in females 1.22–1.34. Coloration varies very slightly, recently moulted specimens are lighter.

#### Distribution and habitat.

The species is known only from the type locality (Qetura) represented by an extra-arid stony desert at 200–500 m above sea level. All specimens were collected with pitfall traps.

## Discussion

### The composition and distribution of the Chediminae

Of the three recognized palpimanid subfamilies, the Palpimaninae was reviewed by Platnick (1981) and the Otiothopinae was revised by Platnick (1975) and Platnick et al. (1999). On the contrary, the Chediminae was briefly surveyed only once, by Simon (1893).

**Figure 51.**
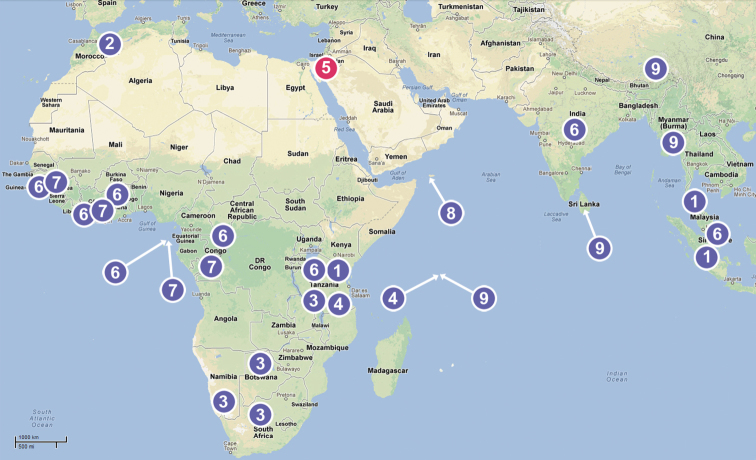
Distribution of genera of the Chediminae: **1** *Boagrius* **2** *Chedima* **3** *Diaphorocellus* **4** *Hybosida* **5** *Levymanus* gen. n. **6** *Sarascelis* **7** *Scelidocteus* **8** *Scelidomachus* **9** *Steriphopus*.

The currently known Chediminae are distributed mainly within the Paleotropical area (Fig. 51). This subfamily includes nine genera: *Badia* Roewer, 1961 (West Africa), *Boagrius* Simon, 1893 (East Africa and South-East Asia), *Chedima* Simon, 1873 (Morocco), *Diaphorocellus* Simon, 1893 (South and South-East Africa), *Hybosida* Simon, 1898 (East Africa, Seychelles), *Sarascelis* Simon, 1887 (tropical parts of Africa and Asia), *Scelidocteus* Simon, 1907 (West Africa), *Scelidomachus* Pocock, 1899 (Socotra) and *Steriphopus* Simon, 1893 (Seychelles and South Asia). Almost all of them are undoubtedly correctly placed in this subfamily, apart from *Badia* Roewer.

**Figure 52.**
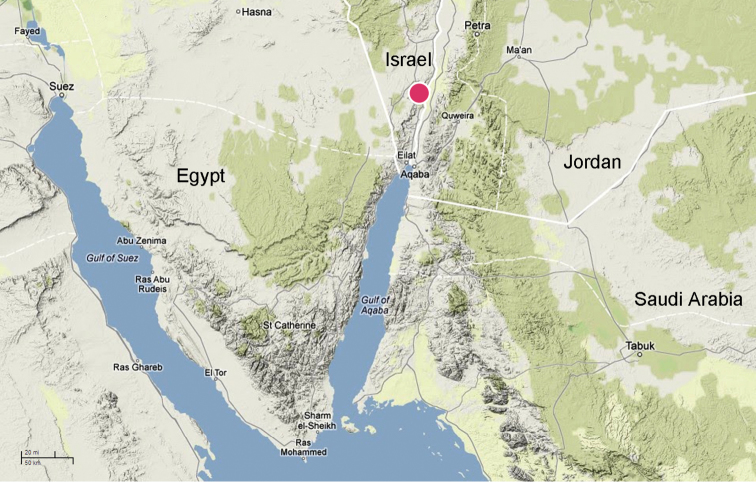
*Levymanus gershomi* sp. n., distribution.

**Figures 53–56.**
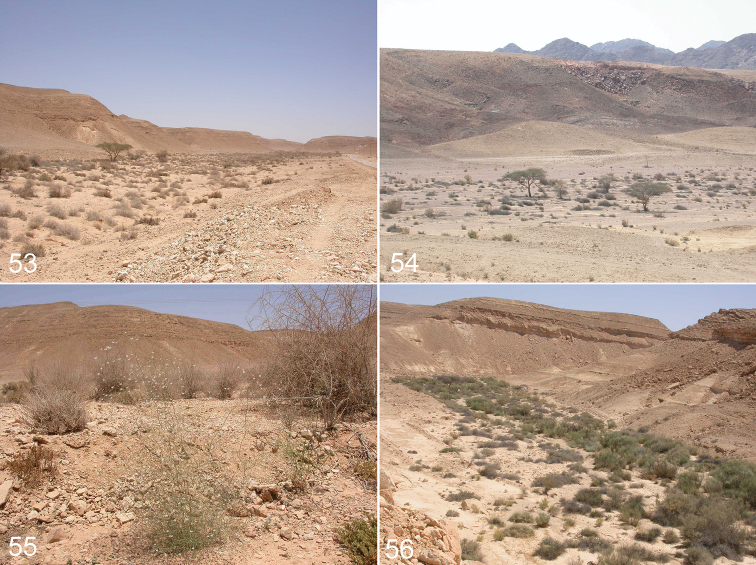
Surroundings of Qetura (Ktura), the type locality of *Levymanus gershomi* sp. n.

Roewer (1961) considered *Badia* as a close relative of *Hermipella* Lessert, 1936 and therefore the former genus was placed in the palpimanid subfamily Hermipellinae Roewer, 1942. Platnick (1989) transferred *Hermipella* to Zodariidae, and later this genus was synonymized with the zodariine genus *Palfuria* Simon, 1910 (Jocqué 1991). Thus, the current placement of *Badia* within Chediminae (Dippenaar-Schoeman & Jocqué 1997) seems to represent a default taxonomic position since it was not included in either Otiothopinae (Platnick 1975; Platnick, Grismado & Ramírez 1999), or in Palpimaninae (Platnick 1981).

The holotype of *Badia rugosa* was not found. Only a single microslide containing a separate leg of this specimen is deposited in SMF (Julia Altmann, personal communication). We thus consider the holotype of *Badia rugosa* to be lost as is the case in several other types from the same study (see Sierwald 1997, Lotz 2007).

According to the original description, *Badia* possesses lateral eyes, ALE and PLE, widely spaced from each other (cf. Roewer 1961, fig. 3a), like in the members of the Palpimaninae. Meanwhile, all chedimine genera recognised by Simon (1893, 1898, 1907) have these eyes (near) contiguous. This feature is considered as one of the key characters of the group and a reliable criterion to distinguish representatives of both subfamilies (see Forster and Platnick 1984, p. 76, fig. 282; Dippenaar-Schoeman and Jocqué 1997, pp. 239, 240, figs 100a, b, d–f). Hence, we conclude that *Badia* should be excluded from the Chediminae and transferred to the Palpimaninae.

The taxonomic position of *Fernandezina gyirongensis* Hu & Li, 1987 from Xizang (Tibet) also appears doubtful. *Fernandezina* Birabén, 1951, the genus to which this species was originally referred is known exclusively from the Neotropical Region, from Guyana to Northern Argentina (Platnick 1975; Platnick et al. 1999). Platnick (1989, 2013) considered this Chinese taxon as certainly misplaced.

The structure of the male bulb in this species, bearing a tegular process (see also Hu 2001, fig. 8–15, 3, 4), does not actually resemble the palps of any *Fernandezina* species (cf. Platnick 1975, figs 86, 87, 90–93; Ramírez and Grismado 1996, figs 1, 2; Platnick et al. 1999, figs 19–24, 29–33; Grismado 2002, figs 2–4; Piacentini et al. 2013, fig. 6c–e). On the contrary, this structure does not differ significantly from that observed in the chedimine genera (cf. Jézéquel 1964, figs 2a, 2b, 4a, 4b, 7a, 7b, 9a, 9b, 11a, 11b).

Within three Oriental genera of the Chediminae, *Boagrius*, *Sarascelis* and *Steriphopus*, the two former genera possess much larger anterior median eyes (see Hu 2001, figs 8–15, 1; Jézéquel 1964, figs 5a–c; Deeleman-Reinold 2001, fig. 76); additionally, in males belonging to these two genera the additional palpal structures are either considerably longer (*Boagrius*) or look more massive (*Sarascelis*) than in *Fernandezina gyirongensis*. Furthermore, the palpal patella in males of *Sarascelis* is more or less hooked and the cymbium is either asymmetrical or with a well-developed lateral process. All these characters are absent in *Fernandezina gyirongensis*.

The third genus, *Steriphopus* (described originally under *Pachypus* Pickard-Cambridge, 1873 *nom. praeocc.*), possesses smaller AMEs (like in *Fernandezina gyirongensis*). Strictly speaking, at the first view other characters noted and figured by Pickard-Cambridge (1873) make *Steriphopus* dissimilar not only to *Fernandezina gyirongensis*, but also to any other palpimanids. According to the original description, the holotype male of *Steriphopus macleayi* (Pickard-Cambridge, 1873), the generotype, has a developed ventral scutum that extends almost to the spinnerets (Pickard-Cambridge 1873, pl. 16, figs 2b, 2c) and an unusually long cymbium which is figured as a structure about three times longer then the palpal tibia (Pickard-Cambridge 1873, pl. 16, fig. 2m). But it should be noted that these described features and the corresponding figures do not reflect the actual characters of the holotype we examined. Contrary to the description, the holotype possesses a moderately sclerotised abdomen and a large sub-globular palpal tibia that appears to be even slightly longer then the cymbium (Figs 8, 9). Other characters of the holotype, including the broad-oval shape of the carapace and configuration of the bulb bear a certain resemblance to *Fernandezina gyirongensis*.

Therefore, the given species is provisionally placed in *Steriphopus* and the new combination is proposed: *Steriphopus gyirongensis* (Hu & Li, 1987), comb. n., with reservation and assumption, that this species may represent a separate chedimine genus, as yet undescribed (since we could not study the holotype, which is lost – Shuqiang Li personal communication).

### Characters and relationships of *Levymanus gershomi* sp. n.

The distinctive characters of the new taxon are listed and discussed below. It should be noted that within the Chediminae some characters, such as the structure of the spermathecae and fine structure of the male palp are known only for a few species described or surveyed after the 1960s (Jézéquel 1964; Platnick 1979; Forster and Platnick 1984). Since we have to exclude these parameters from the comparison, our conclusions are thus preliminary (the putative apomorphies of the new taxon are marked A1–A7).

First and foremost, *Levymanus gershomi* sp. n. differs from all other chedimine palpimanids by having long slender legs (A1) and an elongate body (A2) – as shown in Figs 1–2. All examined members of Chediminae may be referred to the “standard” palpimanid type with a more or less compact or robust body and considerably shorter and thicker legs, as in Figs 3–9. Among other palpimanids, some species of *Fernandezina* (Otiothopinae) also have somewhat longer and thinner legs and a more elongate body (Platnick 1975, figs 80, 85; Grismado 2002, fig. 1). However, the modifications are considerably less strong than in *Levymanus gershomi* sp. n. In addition, species of *Fernandezina* possess a much shorter pedicel tube (cf. Platnick 1975, fig. 88).

As has already been noted, *Levymanus gershomi* sp. n. possesses a thoracic fovea divided into two parts (A3); all other palpimanids have an entire, short fovea that may be longitudinal, transverse, pit-like, or anchor-shaped (Platnick 1975, figs 10, 80; Forster and Platnick 1984, figs 269, 282; Dippenaar-Schoeman and Jocqué 1997, figs 100a, 100b; Platnick et al. 1999, figs 25, 63, 67; Buckup and Ott 2004, figs 1, 2). A bipartite thoracic fovea is characteristic for members of the Stenochilidae, the sister group of palpimanids, but stenochilids possess foveal sulci located longitudinally (cf. Platnick and Shadab 1974, figs 1, 16, 24, 25; Forster and Platnick 1984, figs 308, 310), whereas in *Levymanus gershomi* sp. n. they are located transversely (Figs 10, 12). Hence, in view of this state in the latter species, one of the diagnostic characters of the Palpimanidae given by Forster and Platnick (1984), “the fovea is usually distinct but in contrast to the stenochilids is always single” (op. cit., p. 76), should be reconsidered.

Other characteristic features of the new taxon are edentate chelicerae, lacking the stridulatory ridges (A4), and the considerably reduced scopula (A5) and spinnerets (A6). The presence of stridulatory organs in palpimanids is not well documented. Platnick et al. (1999) showed that most the Otiothopinae generapossess stridulatory files on the chelicerae (op. cit., figs 7, 8, 41, 54–56). Within the Chediminae we have observed similar structures at least in *Boagrius*, *Sarascelis* and *Scelidoucteus* (Zonstein and Marusik in prep.) – i.e., in all available genera represented by large-sized species.

A dense prolateral brush of scopular hairs on the tibia, metatarsus and tarsus of leg I is very characteristic for the whole family Palpimanidae (Forster and Platnick 1984; Jocqué and Dippenaar-Schoeman 2006), though we found that in *Steriphopus macleayi* it seems to be less developed than in other examined palpimanids (cf. Figs 3–9). However, in *Levymanus gershomi* sp. n. the scopula is even weaker and represented only by sparsely distributed spatulate hairs (Figs 26–31).

Although all palpimanids possess strongly reduced spinnerets (Forster and Platnick 1984; Jocqué and Dippenaar-Schoeman 2006), in *Levymanus gershomi* sp. n.this reduction is extreme, and only tiny mound-shaped ALS are visible both in male and female (as in Figs 32, 34). In all other members of Chediminae the ALS are conical to cylindrical and well-discernible (Figs 4, 6–8; Platnick 1979, figs 1, 3, 4; Hu 2001, fig. 2).

The structure of the palp in the new taxon does not differ strongly from that in other members of the subfamily. The most significant distinction is the presence of a tegular furrow (A7) (Figs 38, 40, 41, 43), a detail that has not been found in any other examined genera of the chedimine palpimanids.

We thus conclude that *Levymanus* gen. n. is distinct from all other genera within the subfamily. Moreover, the above-noted characters contrast with all other genera of Chediminae taken as a whole. Currently it is not certain whether this new taxon represents a separate subfamily or whether itshould be considered only as a specialized chedimine palpimanid. This question might be answered in the course of a taxonomic revision and phylogenetic study of the Chediminae.

## Supplementary Material

XML Treatment for Levymanus

XML Treatment for Levymanus gershomi
